# Mineral–nutrient relationships in African soils assessed using cluster analysis of X-ray powder diffraction patterns and compositional methods

**DOI:** 10.1016/j.geoderma.2020.114474

**Published:** 2020-10-01

**Authors:** Benjamin M. Butler, Javier Palarea-Albaladejo, Keith D. Shepherd, Kamau M. Nyambura, Erick K. Towett, Andrew M. Sila, Stephen Hillier

**Affiliations:** a The James Hutton Institute, Craigiebuckler, Aberdeen AB15 8QH, UK; b Biomathematics & Statistics Scotland, JCMB, The King’s Buildings, Edinburgh EH9 3FD, UK; c World Agroforestry Centre (ICRAF), P.O. Box 30677-00100 GPO, Nairobi, Kenya; d International Fertiliser Development Centre, c/o icipe Campus, P.O. Box 30772-00100, Nairobi, Kenya; e Department of Soil and Environment, Swedish University of Agricultural Sciences (SLU), SE-75007 Uppsala, Sweden

**Keywords:** Macro-nutrients, Micro-nutrients, Soil mineralogy, X-ray powder diffraction, Cluster analysis, Compositional data analysis

## Abstract

Soil mineral compositions are often complex and spatially diverse, with each mineral exhibiting characteristic chemical properties that determine the intrinsic total concentration of soil nutrients and their phyto-availability. Defining soil mineral-nutrient relationships is therefore important for understanding the inherent fertility of soils for sustainable nutrient management, and data-driven approaches such as cluster analysis allow for these relations to be assessed in new detail.

Here the fuzzy-c-means clustering algorithm was applied to an X-ray powder diffraction (XRPD) dataset of 935 soils from sub-Saharan Africa, with each diffractogram representing a digital signature of a soil’s mineralogy. Nine mineralogically distinct clusters were objectively selected from the soil mineralogy continuum by retaining samples exceeding the 75% quantile of the membership coefficients in each cluster, yielding a dataset of 239 soils. As such, samples within each cluster represented mineralogically similar soils from different agro-ecological environments of sub-Saharan Africa. Mineral quantification based on the mean diffractogram of each cluster illustrated substantial mineralogical diversity between the nine groups with respect to quartz, K-feldspar, plagioclase, Fe/Al/Ti-(hydr)oxides, phyllosilicates (1:1 and 2:1), ferromagnesians, and calcite.

Mineral–nutrient relationships were defined using the clustered XRPD patterns and corresponding measurements of total and/or extractable (Mehlich-3) nutrient concentrations (B, Mg, K, Ca, Mn, Fe, Ni, Cu and Zn) in combination with log-ratio compositional data analysis. Fe/Al/Ti/Mn-(hydr)oxides and feldspars were found to be the primary control of total nutrient concentrations, whereas 2:1 phyllosilicates were the main source of all extractable nutrients except for Fe and Zn. Kaolin minerals were the most abundant phyllosilicate group within the dataset but did not represent a nutrient source, which reflects the lack of nutrients within their chemical composition and their low cation exchange capacity. Results highlight how the mineral composition controls the total nutrient reserves and their phyto-availability in soils of sub-Saharan Africa. The typical characterisation of soils and their parent material based on the clay particle size fraction (i.e. texture) and/or the overall silica component (i.e. acid and basic rock types) alone may therefore mask the intricacies of mineral contributions to soil nutrient concentrations.

## Highlights

• Cluster analysis applied to soil X-ray powder diffraction patterns.
• Nine mineralogically distinct clusters of soils defined.
• Statistically significant differences in nutrient compositions between clusters.
• Feldspars and Fe/Ti/Al/Mn-(hydr)oxides drive total nutrient concentrations.
• 2:1 phyllosilicates drive extractable (Mehlich-3) nutrient concentrations.

## 1 Introduction

Minerals are the major component of most soils. Through direct inheritance from the parent material and subsequent alteration by chemical weathering, the soil mineral composition can be spatially diverse - reflecting the many soil forming factors (Jenny, 1994). Minerals present within the soil environment exhibit characteristic crystal structures, chemical compositions and chemical properties (Dixon and Schulze, 2002) that determine the total reserves of essential plant nutrients (White and Brown, 2010). Aside from these total nutrient reserves, the fate, phyto-availability and toxicity of these nutrients depends upon the form in which they occur (Cornu et al., 2009) along with associated dissolution–precipitation, adsorption–desorption and reduction–oxidation reactions (Singh and Schulze, 2015).

Soil macro- and micro-nutrient concentrations and their phyto-availability are inherently related to soil fertility (Ajiboye et al., 2019). Understanding mineral contributions to soil nutrient reserves is a key priority at present given the threat of negative soil nutrient balances to food security (Sutton et al., 2013, Omuto and Vargas, 2018). Further, estimates suggest that 60% of cultivated soils suffer from growth-limiting problems relating to nutrient deficiencies and/or toxicities (Cakmak, 2002, Schjoerring et al., 2019, Keskinen et al., 2019). In particular, many African soils are considered to have a typically low fertility caused by a lack of volcanic/tectonic rejuvenation, resulting in long and repeated cycles of weathering, erosion and leaching predominating over millions of years. This leaves the soils poor in nutrients, especially soils derived from basement complex rocks and aeolian sands (Voortman et al., 2003). Furthermore, since the use of fertiliser in sub-Saharan African crop production is very limited, there is a dependence on inherent reserves of soil nutrients (Vitousek et al., 2009, Vanlauwe et al., 2014, Keskinen et al., 2019), of which mineralogy is a key component.

Indeed, soil fertility degradation has been described as the single most important constraint on food security in sub-Saharan Africa (Smaling, 1994). By 2050, the population of sub-Saharan Africa is expected to increase 2.5-fold and demand for cereals approximately triple (Van Ittersum et al., 2016). To maintain the current level of cereal self-sufficiency of approximately 80% by 2050, nearly complete closure of the gap between current farm yields and water-limited yield potential is needed, which is in the range of 20% to 50% (Van Ittersum et al., 2016). Meeting these demands for food production will require an increase in nutrient inputs combined with a better understanding of the intrinsic soil mineral reserves of plant nutrients.

X-ray powder diffraction (XRPD) measurements are commonly used to identify and quantify soil mineralogy because diffraction data are fundamentally related to the crystal structure and crystal chemistry of the soil minerals present (Schulze, 1989). A typical soil XRPD pattern (diffractogram) is comprised of discrete ‘Bragg’ diffraction peaks varying in intensity (y) that are distributed along an experimental axis (x) usually expressed in degrees 2θ. The ‘Bragg’ peaks rise above a background that often includes diffuse scattering from X-ray amorphous components such as soil organic matter or volcanic glass. The mineralogical detail associated with soil XRPD analysis therefore makes it arguably the most powerful approach to accurately identify and quantify the complex suite of minerals in the soil environment. Classically, identifying and quantifying soil minerals from XRPD data is a time and labour intensive process, requiring each diffractogram to be manually inspected and analysed in combination with mineral databases [e.g. the Powder Diffraction File; ICDD, 2019] and specialised computer software. Such workflows become inconvenient when processing large numbers of samples, and the recent acquisition of high-throughput XRPD datasets containing thousands of soil diffractograms has promoted application of alternative, data-driven, approaches to soil XRPD data for the first time (Butler et al., 2018, Butler et al., 2019, Hillier and Butler, 2018). These approaches remove the need for classical expert interpretation in the initial stages of analysis, which can be particularly challenging and time consuming in diverse datasets of this size.

One such high-throughput soil XRPD dataset is that of the Africa Soil Information Service (AfSIS) Sentinel sites, which contains approximately 2000 georeferenced soil samples from sub-Saharan Africa that have each been analysed by XRPD (Vågen et al., 2010, Towett et al., 2013). Associated with each XRPD measurement is a range of site attributes and soil property measurements, including total and extractable nutrient concentrations. By treating each diffractogram within this dataset as a reproducible mineralogical signature of a soil, the data can be combined with data-driven analysis and related to the associated nutrient concentrations in order to identify and interpret mineral contributions to soil nutrient concentrations. The application of data-driven analysis to soil XRPD data defines a new concept for soil mineralogy research which has been labelled ‘Digital Soil Mineralogy’ (Hillier and Butler, 2018).

Cluster analysis is a branch of multivariate statistical analysis for unsupervised machine learning and has notable potential to facilitate expert interpretation of soil mineralogy–nutrient relationships in large XRPD datasets by grouping the data into a manageable number of classes. Each cluster defined from soil XRPD data should represent a group of mineralogically similar soils that are mineralogically distinct from those in other clusters. Recently, a suitable protocol for clustering soil XRPD data has been determined (Butler et al., 2019), and the present study uses this protocol to investigate mineral–nutrient relationships in soils sampled from a diverse range of agro-ecological environments in sub-Saharan Africa (Vågen et al., 2010). Analysis includes a suite of macro-nutrients (Mg, K and Ca) and micro-nutrients (B, Mn, Fe, Ni, Cu and Zn), both in terms of total and extractable concentrations. Novel statistical approaches are applied that account for the compositional nature of nutrient concentration data. That is, multivariate data conveying relative information, with values of the constituting components representing fractions of a total (e.g. 100 or $10^{6}$ when expressed respectively in percentage or ppm units). Such data are only meaningful in relation to each other, regardless of the measurement scale used, and compositional data analysis methods seek to ensure that meaningful conclusions are obtained on this basis (Aitchison, 1982). In combining soil XRPD patterns and nutrient concentrations with cluster analysis and compositional methods, we generate new understanding that can contribute to a move towards more sustainable and mineralogically tailored land management practices.

## 2 Sample collection and laboratory analyses

### 2.1 Soil sampling

**Fig. 1:**
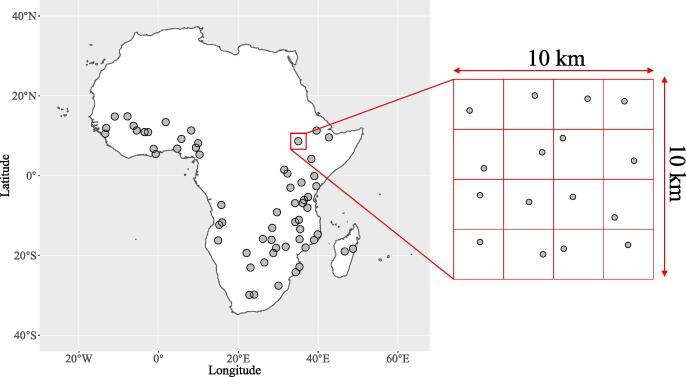
The survey design used for the sampling of Sentinel sites. Each of the sixty 10×10 km sites was separated into a 4×4 grid, and a soil at a random location within each grid cell sampled.

Georeferenced samples associated with the AfSIS project (Vågen et al., 2010) were taken from the set of sixty 10×10 km ‘Sentinel’ sites distributed across sub-Saharan Africa (Fig. 1). The Sentinel sites were designed to be statistically representative of the variability in climate, topography and vegetation of sub-Saharan Africa (Vågen et al., 2010, Hengl et al., 2017). Field sampling was conducted based on the field methods collectively referred to as the Land Degradation Surveillance Framework (LDSF) protocol (Vågen et al., 2010, Vågen et al., 2015). Each Sentinel site was divided into 16 grid cells of equal size, and a soil sampled at a random location within each grid cell (Fig. 1).

Only subsoil samples were investigated here with the aim of minimising the potential effects of land management practices (see Table 1 in Towett et al., 2015) and soil organic matter on nutrient concentrations, both of which would be more prominent in topsoils. In doing so the naturally occurring mineral contributions to soil element concentrations could be focused upon. Subsoil samples were collected at depths of 20-50 cm from each grid cell at each Sentinel site, giving a total of 960 soil samples (16 grid cells per site × 60 Sentinel sites) of which 935 were measured by XRPD.

**Table 1:** Soil property summary statistics. Each property is summarised by its 10th, 25th, 50th, 75th and 90th percentiles for the whole dataset (n=935). Clay represents the clay particle size fraction (<2μm). All data are summarised by their geometric mean and geometric standard deviation except for pH, where the arithmetic mean and arithmetic standard deviation are used. The geometric standard deviation is related to the geometric mean by multiplication and division, represented by ‘⋇’. The arithmetic standard deviation is related to the arithmetic mean by addition and subtraction, represented by ‘±’. All concentrations are in units of mg kg^−1^ except for pH and Clay (<2μm particle size fraction; % by volume).

	Percentile, all soils						Mean and standard deviation								
Property	10th	25th	50th	75th	90th		Cluster 1	Cluster 2	Cluster 3	Cluster 4	Cluster 5	Cluster 6	Cluster 7	Cluster 8	Cluster 9

pH	4.94	5.35	6.01	6.69	8.13		6.10±0.99	5.56±1.07	5.39±0.56	5.87±0.80	5.82±0.76	6.60±1.29	6.02±0.91	8.02±0.90	5.86±0.52
Clay (%)	12.42	23.88	43.21	67.26	78.77		10.89⋇2.15	29.21⋇1.38	55.78⋇1.27	25.09⋇1.45	61.19⋇1.25	47.43⋇1.45	70.7⋇1.2	73.4⋇1.18	77.75⋇1.14
															
TOC	2178	3113	5489	11139	20176		2089⋇1.71	3593⋇1.77	7137⋇1.79	4134⋇1.73	8588⋇2.11	6473⋇2.13	12813⋇1.96	10503⋇1.70	23054⋇2.41
K_T_	1117	2482	7046	16948	26556		2505⋇2.27	2530⋇2.75	2890⋇1.98	28819⋇1.36	5861⋇2.23	13877⋇1.78	6088⋇3.35	21573⋇1.32	4516⋇1.89
Ca_T_	183	400	1254	5028	19873		342⋇2.14	277⋇2.75	479⋇1.97	1844⋇2.23	1165⋇2.14	3796⋇2.37	1842⋇4.33	19558⋇2.33	3071⋇2.71
Mn_T_	36	96	216	530	1029		38⋇2.23	60⋇3.10	173⋇2.07	170⋇1.69	339⋇2.13	457⋇1.59	721⋇2.20	558⋇1.91	1105⋇2.01
Fe_T_	3518	8714	20675	36643	61561		3020⋇1.78	3525⋇5.65	19759⋇1.29	11161⋇1.49	36032⋇1.29	26140⋇1.34	54828⋇1.41	36489⋇1.17	90529⋇1.33
Ni_T_	2	5	13	25	47		3⋇2.27	3⋇2.56	12⋇1.81	5⋇2.21	25⋇1.51	18⋇1.50	26⋇2.14	72⋇2.34	37⋇1.88
Cu_T_	3	5	12	20	31		3⋇1.77	4⋇2.08	12⋇1.87	6⋇1.51	21⋇1.46	17⋇1.45	25⋇1.57	21⋇1.22	29⋇1.88
Zn_T_	4	8	19	35	59		4⋇1.97	6⋇1.84	19⋇1.63	12⋇1.45	22⋇1.57	30⋇1.69	40⋇1.67	55⋇1.32	52⋇1.65
															
B_M_	0.001	0.001	0.081	0.310	0.829		0.003⋇11.61	0.002⋇10.66	0.027⋇11.93	0.013⋇16.09	0.064⋇6.85	0.106⋇14.64	0.133⋇7.34	2.265⋇1.87	0.044⋇9.65
Mg_M_	39	71	150	386	815		79⋇1.99	53⋇2.40	82⋇1.87	129⋇1.63	255⋇2.60	331⋇1.73	273⋇3.11	1414⋇1.64	335⋇2.35
K_M_	29	45	77	158	334		51⋇2.32	52⋇2.15	58⋇1.85	84⋇2.38	89⋇1.76	157⋇2.60	91⋇2.12	677⋇1.96	115⋇2.27
Ca_M_	122	251	570	1540	5104		279⋇2.08	176⋇2.97	315⋇2.28	521⋇1.93	782⋇2.25	1352⋇2.22	881⋇3.44	7050⋇2.48	1433⋇2.38
Mn_M_	5	18	62	130	334		17⋇3.05	18⋇5.71	37⋇3.40	37⋇2.65	62⋇3.95	137⋇1.94	111⋇3.66	83⋇4.30	55⋇3.70
Fe_M_	40	58	86	126	175		72⋇1.92	101⋇1.54	95⋇1.61	95⋇1.60	101⋇1.45	91⋇1.71	102⋇1.47	43⋇2.02	102⋇1.57
Cu_M_	0.2	0.5	1.1	2.4	3.8		0.1⋇4.62	0.4⋇4.15	1.0⋇2.69	0.8⋇1.68	2.1⋇1.87	2.6⋇1.54	2.2⋇2.13	3.0⋇1.33	1.9⋇2.57
Zn_M_	0.4	0.6	1.0	1.5	2.4		0.8⋇1.65	0.9⋇1.62	1.0⋇1.80	0.6⋇8.3	1.0⋇2.05	1.3⋇1.89	1.1⋇2.03	1.0⋇1.88	0.8⋇2.40

### 2.2 Laboratory analyses

A total of 10 elements are examined herein to understand how nutrient and micro-nutrient concentrations relate to the mineralogy of African soils. These elements include:

1. The total organic carbon (TOC) concentration, used as an estimate of soil organic matter content.
2. Mg, K and Ca concentrations examined as essential plant macro-nutrients.
3. B, Mn, Fe, Ni, Cu and Zn concentrations examined as essential plant micro-nutrients.

Further to these 10 elements, soil pH and clay particle size fraction were also examined as common soil properties linked to plant nutrient provision. All laboratory analyses were conducted on the dry sieved <2 mm size fraction.

#### 2.2.1 Determination of total organic carbon, pH and clay particle size fraction

Samples were prepared for TOC analysis by acidification with hydrochloric acid and drying at 60 °C using a modification of the protocol described in Harris et al., 2001. The carbon concentrations were then determined by thermal oxidation on a Thermoquest FlashEA 1112 equipped with an autoanalyser (Vågen et al., 2010).

Soil pH was determined with a combination electrode on the supernatant of a 1:2 soil:solution ratio. Clay particle size fraction was determined by laser diffraction as the percentage volume of <2μm sized particles (assuming spherical morphology) on a Horiba LA-950V2 Particle Size Analyser with a detectable size range of 0.01-3000μm (Vågen et al., 2010). It is important to distinguish that the clay particle size fraction includes any mineral particle <2μm in diameter. Whilst this size delineation is most often associated with the clay minerals (i.e. phyllosilicates), this does not rule out the fact that other rock forming minerals can sometimes be present in the clay particle size fraction, and conversely phyllosilicates can have particle sizes >2μm. As such the clay particle size fraction may differ to the percentage of phyllosilicates/clay minerals determined from quantitative mineralogical analysis (Section 2.2.3).

#### 2.2.2 Determination of nutrient concentrations

Total X-ray fluorescence spectroscopy (TXRF) was used to determine the total concentrations of K, Ca, Mn, Fe, Ni, Cu, and Zn (hereafter denoted by subscript ‘T’, e.g. K_T_). Detailed accounts of the TXRF protocol and limits of detection are provided in Towett et al., 2013, Towett et al., 2015. Briefly, 50 mg of air-dried (40 °C) and ground (20–50 μm) soil sample was mixed with 2.5 ml of Triton X100 (Fisher) solution (0.1 vol.%) to form a soil suspension, and spiked with 40 μl of 1000 mg l^−1^ Selenium (Fluka) as the internal standard. The resulting suspension was placed into an ultrasonic water bath at room temperature and sonicated for 15 min, and then mixed well using a digital shaker. 10 μl of the turbid soil solution was then dispensed onto a clean siliconised quartz glass sample carrier and dried for 10-15 min at 52 °C. Samples were analysed in triplicate on an S2 PICOFOX TXRF (Bruker) with a data acquisition time of 1000 s per sample. Spectral evaluation and element quantification were performed using the software SPECTRA 6.3 (Bruker).

Phyto-available nutrient concentrations were estimated using the Mehlich-3 extraction protocol (Mehlich, 1984, Ziadi and Tran, 2007), hereafter referred to as ‘M3’. The M3 extracting solution is composed of 0.2 N ethanoic acid (CH_3_COOH), 0.25 N ammonium nitrate (NH_4_NO_3_), 0.015 N ammonium fluoride (NH_4_F), 0.013 N nitric acid (HNO_3_), and 0.001 M ethylenediaminetetraacetic acid (C_10_H_16_N_2_O_8_). The method allows for multiple elements to be analysed from a single extraction, namely B, Mg, K, Ca, Mn, Fe, Cu and Zn in this case - hereafter denoted by subscript ‘M’, e.g. K_M_. All extracted elements were quantified by inductively-coupled plasma optical emission spectroscopy using a Perkin Elmer Optima 8300 instrument.

#### 2.2.3 X-ray powder diffraction and mineral quantification

Sub-sampling for XRPD analysis was conducted by coning and quartering. Sub-samples were then prepared for XRPD by McCrone milling 3 g of sieved (<2 mm) and air-dried soil for 12 min in ethanol. Excess ethanol was removed by centrifugation and each sample re-suspended in 1.5 ml of hexane. The samples were then oven dried at 80 °C before being disaggregated and ground by hand in a mortar and pestle before passing through a 250 μm sieve. Loading into the instrument was carried out by loosely filling the sample holders with the finely ground powders, before flattening the surface with the sharp edge of a razor blade, with personnel instructed to take care to apply minimum pressure and to avoid shearing motion. The combination of methodical milling and loading was designed to produce samples with appropriate particle statistics and to minimise preferred orientation (Zhang et al., 2003).

After loading, XRPD data were collected on a Bruker desktop D2 PHASER diffractometer, with Ni-filter, Cu-Kα radiation with the X-ray tube operated at 30 kV and 10 mA. The beam was collimated using a 0.6 mm divergence slit, a 1 mm anti-scatter slit and a 2.5 mm Soller slit. Samples were rotated continuously at 15 rpm during data collection over the angular range of 3 to $75{\;}^{\circ }2\theta$, counting for 96 s per $0.02^{\circ }$ step with a Lynxeye position sensitive detector.

To quantitatively summarise the mineralogy of each cluster, untreated diffractograms were aligned relative to a standard quartz pattern (ICDD, 2019, Butler et al., 2019), and the mean diffractogram of each cluster computed. Phases in each mean diffractogram were identified using the Powder Diffraction File database (ICDD, 2019), taking all peaks of each mineral component into account, and subsequently quantified by the full pattern summation method (Omotoso et al., 2006) as implemented in the ‘powdR’ package (Butler and Hillier, 2020) of the R language and environment for statistical computing (R Core Team, 2018). This approach treats an observed diffractogram as the sum of contributions from individual crystalline, para-crystalline and amorphous components within it. Using a library of prior measured diffractograms of pure phases (Eberl, 2003) and their Reference Intensity Ratios (Hillier, 2000), an observed pattern can be modelled as the sum of these pure components and their concentrations can be accurately determined (Omotoso et al., 2006). Given the overlapping features of some clay minerals in bulk XRPD measurements (i.e. randomly oriented milled subsamples of the <2 mm fraction), clay minerals characterised as dioctahedral smectites along with mixed-layered (interstratified) clay minerals with dioctahedral character were grouped into a general category labelled as ‘dioctahedral expandable’ phyllosilicates.

## 3 Statistical analyses

All statistical analyses outlined in subsequent sections were conducted using the R language and environment for statistical computing (R Core Team, 2018). Statistical test results were assessed at the usual 0.05 significance level.

### 3.1 Cluster analysis of X-ray powder diffraction data

Prior to cluster analysis, the XRPD data were subset to the $6-75{\;}^{\circ }2\theta$ range before being pre-treated by alignment, binning (bin width = 5), square-root transformation and mean centering as recommended by Butler et al., 2019. This procedure was designed to stabilise sample independent variability of the signals to enhance comparison across samples, whilst reducing the overwhelming signal from strong diffractors such as quartz.

For the cluster analysis, principal component analysis [PCA, Jolliffe, 1986] was firstly applied to reduce the pre-processed XRPD dataset by projection onto a low-dimensional space (Rossel et al., 2016, Butler et al., 2019). Principal components that explained 99% of pre-processed data variability (in this case 21 principal components) were then used as the input for the fuzzy-c-means clustering algorithm (Bezdek et al., 1984, Meyer et al., 2017). Fuzzy clustering allows for classification uncertainty by considering soft boundaries between clusters so that for each sample it determines membership coefficients, defined in the [0,1] interval, to each cluster (using a cluster fuzziness hyper-parameter set to 2). The optimum number of clusters was objectively derived by applying the fuzzy-c-means algorithm to 19 iterations with cluster nodes ranging from 2 to 20, and selecting the instance with the highest partition coefficient from the clustering statistics (Bezdek et al., 1984, Rossel et al., 2016). To facilitate neater characterisation of clusters from the soil mineralogy continuum, only samples exceeding the 75% quantile of the membership coefficients in each cluster were retained for further inspection of soil properties across the clusters. This yielded a subset of the data containing 239 samples upon which all subsequent analysis was applied, allowing the investigation to focus on more distinct mineralogical groupings within the data.

### 3.2 Compositional analysis of nutrient concentrations

Nutrient concentrations describing the relative make-up of a soil sample are inherently multivariate data. Regardless of the units of measurement, the data are constrained in that each chemical species measurement is a fraction or part of a total (e.g. total sample weight or volume) and, hence, changes in the relative abundance of one species necessarily implies changes in at least one of the others. It has been long recognised that, for example, ordinary correlation measures computed on data carrying relative information can result in spurious associations, with pairwise correlations between parts not being consistent when measured from a full composition or a subset of it (Pearson, 1897, Aitchison, 1986). Since the correlation structure of a dataset is a key piece of information in multivariate analysis, this is a fundamental drawback. Following on the seminal work by Aitchison, 1986, the mainstream approach to dealing with compositional data focuses on the analysis of log-ratios between parts which contain the relative information. This eliminates technical issues like spurious correlations, singularity of the covariance matrix or data curvature, producing results which do not depend on the scale of the data nor the sample total. Moreover, through log-ratios the data are mapped onto the ordinary real space, which then facilitates analysis, modelling and visualisation using ordinary statistical methods on real-valued log-ratio coordinates.

A form of log-ratios, so called balances (Egozcue and Pawlowsky-Glahn, 2005), were used here to represent the total and M3 nutrient compositions (TOC–K_T_–Ca_T_–Mn_T_–Fe_T_–Ni_T_–Cu_T_–Zn_T_ and B_M_–Mg_M_–K_M_–Ca_M_–Mn_M_–Fe_M_–Cu_M_–Zn_M_, respectively, expressed in units of mg kg^−1^; see Section 2.2.2). This facilitated interpretability since these balances correspond to trade-offs between subsets of parts of the composition determined according to their co-dependence structure (measured in terms of proportionality by the variation matrix; see details on the construction of these balances in accompanying Supplementary Material).

#### 3.2.1 Values below the detection limit and average nutrient concentrations

The subset (n=239) M3 dataset included overall 5.23% cells with values below the detection limit, which were mostly concentrated in B_M_ (34.31%) and Cu_M_ (6.69%). These values were dealt with using statistical imputation to enable subsequent multivariate analysis. The log-ratio expectation–maximisation (EM) algorithm (Palarea-Albaladejo et al., 2007) implemented in the ‘zCompositions’ R package (Palarea-Albaladejo and Martin-Fernandez, 2015) was used for this which preserves the log-ratios between parts while accounting for the corresponding detection limit thresholds and the co-dependence structure of the observed data.

Following imputation of values below the detection limit, geometric means were used to summarise the various nutrient concentrations of each cluster (Section 3.1) in a way that is consistent with their relative scale (Aitchison, 1986, Montero-Serrano et al., 2010). It is worth noting that geometric means and associated geometric standard deviations are related to one-another by division and multiplication, as opposed to the traditional subtractions and additions of their arithmetic equivalents. Thus the ‘divide on times’ notation (⋇) is provided wherever geometric means and geometric standard deviations are reported.

#### 3.2.2 Multivariate analysis of variance

The entire nutrient compositions from the total and M3 datasets were statistically compared between the groups determined by cluster analysis of the XRPD data (Section 3.1) using multivariate analysis of variance (MANOVA) on log-ratio balances. More specifically, the non-parametric permutational MANOVA (PERMANOVA) introduced by Anderson, 2001 was applied since the ordinary assumption of multivariate normality for the standard MANOVA was not met. Results from PERMANOVA are invariant to the log-ratio balance representation chosen, as determined by a sequential binary partition into subsets of parts of the composition (Egozcue and Pawlowsky-Glahn, 2005), thus balances built according to co-dependences between parts were used as described in the Supplementary Material.

#### 3.2.3 Principal component analysis biplots

To visualise relationships between log-ratio nutrient balances and soil mineralogy, PCA biplots were produced by PCA of the pre-treated and subset XRPD data (n=239). Log-ratio balances of the total and M3 datasets were provided as supplementary variables to the PCA (Lê et al., 2008) so that they could subsequently be added as vectors to biplots based on the first three principal components. Mineral contributions to the principal components derived from the XRPD data were interpreted using the loadings of each component in combination with the known diffraction features of soil mineral components (ICDD, 2019).

### 3.3 Analysis of variance of soil pH

Soil nutrient concentrations are directly and indirectly related to soil pH via the effects of changing H^+^ activity on adsorption–desorption and dissolution–precipitation reactions (Dixon and Schulze, 2002, Singh and Schulze, 2015). Thus measurements of pH are included within this investigation to help isolate mineralogical drivers of soil nutrient concentrations. The variation in pH between the mineralogically defined clusters (Section 3.1) was assessed using analysis of variance (ANOVA).

## 4 Results and discussion

### 4.1 Cluster analysis

**Fig. 2:**
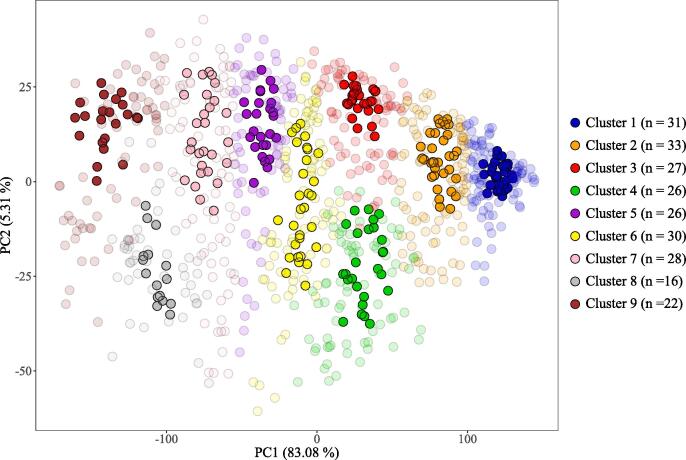
Principal component scores of pre-treated X-ray power diffraction data and the resulting clusters defined by the fuzzy-c-means algorithm. Total number of samples =935. Samples excluded from subsequent analysis are plotted as translucent symbols, whilst those retained are plotted as opaque symbols.

The optimum number of clusters for the XRPD dataset, defined by maximisation of the partition coefficient of the fuzzy-c-means algorithm (Section 3.1), was found to be nine. A broad spread of the XRPD data when plotted in principal component space was observed, reflecting the soil mineralogy continuum (Fig. 2). Certain samples from this continuum were excluded from subsequent analysis (based on membership coefficients <75% quantile threshold; Section 3.1), creating mineralogically discrete clusters more suitable for identifying mineral–nutrient relationships. Estimates of the quartz concentration for each cluster, derived from the quantitative mineral analysis (Sections 2.2.3, 4.1.1), were used to order the clusters from 1 through to 9 by decreasing quartz concentration, and thus aid interpretation of the data. The resulting dataset comprised a total of 239 soil samples across the nine clusters, with cluster sizes ranging from 17 to 33 (Fig. 2).

**Fig. 3:**
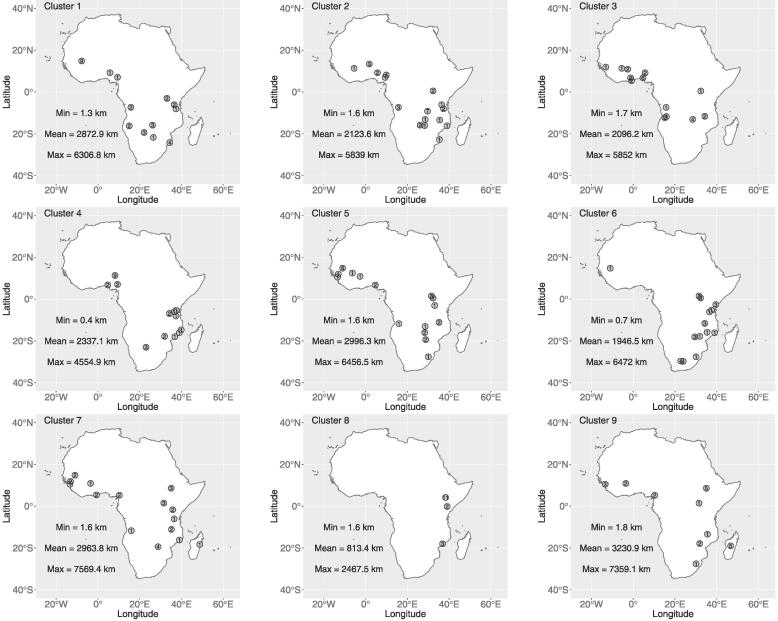
The spatial distribution of soil samples within each cluster. Symbols are labelled according to the number of samples at a given Sentinel site. Min, Mean and Max represent the minimum, mean and maximum distance between soil samples within the cluster, respectively.

The 239 samples in the nine clusters represented soils from 57 of the 60 Sentinel sites in total. The samples in each cluster display considerable spatial variation across sub-Saharan Africa (Fig. 3). The mean number of sites represented by each cluster is twelve, with mean distances between samples within each cluster being approximately 2000-3000 km. The only obvious exception to this is Cluster 8, which is represented by only three sites, with a mean distance between samples of 813 km. Given that the Sentinel sites cover the major agro-ecological zones of sub-Saharan Africa (Vågen et al., 2010, Hengl et al., 2017), the spatial distribution of the clustered data suggests that the mineralogically similar soils within each cluster are representative of a wide variety of land uses, climatic properties and ecosystems (see Table 1 in Towett et al., 2015).

Soil TOC concentration (i.e. organic matter), pH and clay particle size fraction can affect soil nutrient concentrations, and are therefore taken into account here when interpreting the results from the cluster analysis (Table 1). The average pH values for each Cluster range from 5.39 (Cluster 3) to 8.02 (Cluster 8). Notably, the mean pH of Cluster 8 is close to the 90th percentile of the entire dataset (Table 1), thus the soils within this group are relatively unusual in this context. Indeed, ANOVA of pH (Section 3.3) between the clusters yields a statistically significant difference in means only when Cluster 8 is included, suggesting that pH should only be taken into account when interpreting results associated with this cluster. The average clay particle size fraction and soil TOC concentration of the nine clusters both display a general increase from Cluster 1 through to Cluster 9 (Table 1), with notable features that will be discussed in relation to soil mineralogy and nutrient concentrations below.

#### 4.1.1 Soil mineralogy and nutrient concentrations

**Fig. 4:**
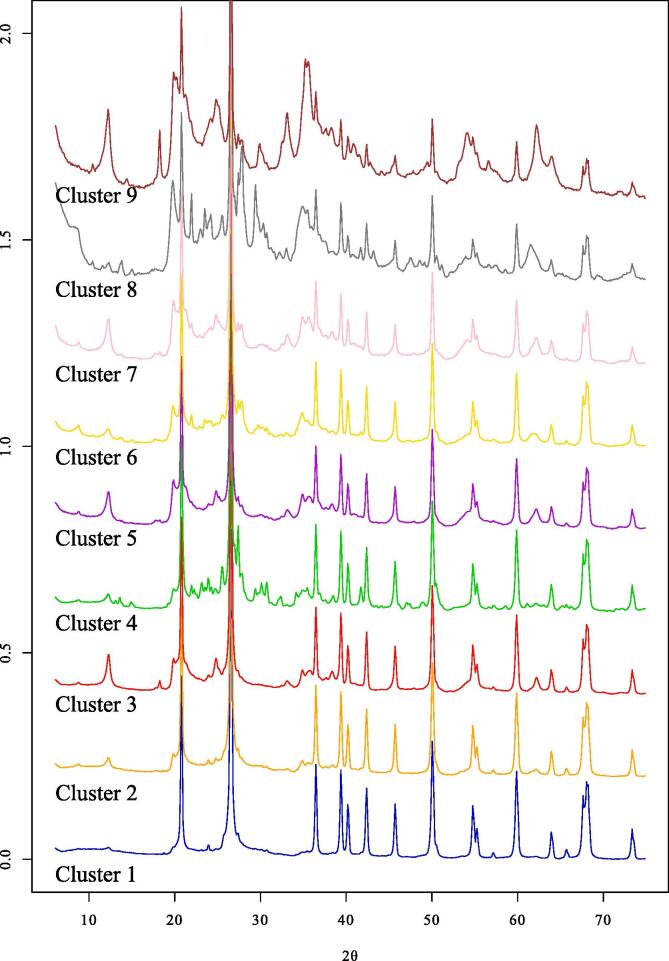
The mean diffractograms for each cluster, which were subsequently used to derive quantitative estimates of each cluster’s mineralogy. The y-axis has been square-root transformed to aid with comparison.

**Fig. 5:**
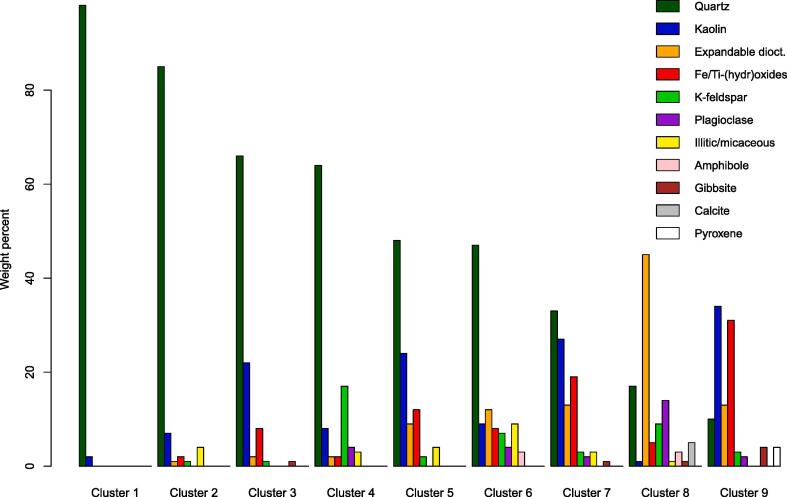
Simplified mineralogy of each cluster, with Fe/Ti-(hydr)oxides grouped together. The Fe/Ti-(hydr)oxide bar therefore represents the sum of goethite, maghemite, ilmenite, hematite and magnetite. A more detailed summary is provided in Table 2.

Quantitative estimates for the mineral compositions of each cluster, derived from their mean diffractograms (Fig. 4), are expressed in terms of weight percent of soil mineral components (i.e. organic matter is not implicit in the composition; Table 2; Fig. 5). This quantitative information allows for improved interpretation of mineral–nutrient relationships from the cluster analysis.

**Table 2:** Quantitative mineralogy estimated from the mean diffractogram of each cluster (Fig. 4). Kaol. = kaolin minerals; Exp. dioct. = expandable dioctahedral minerals; Ill. = illitic/micaceous minerals; K-fel. = K-feldspar minerals; Plag. = plagioclase minerals; Goet. = goethite; Magh. = maghemite; Ilm. = ilmenite; Hem. = hematite; Gibb. = gibbsite; Magn. = magnetite; Anat. = anatase; Amph. = amphibole minerals; Pyrox. = pyroxene minerals; Cal. = calcite; Gyps. = gypsum; ∑Phyllo. = summed concentration of phyllosilicate minerals (i.e. kaolin and 2:1 phyllosilicates); ∑Fe/Al/Ti-(hydr)ox. = summed concentration (excluding values <1%) of Fe/Al/Ti-(hydr)oxide minerals.

				2:1 phyllosilicates			Feldspars			Fe/Al/Ti-(hydr) oxides								Ferromagnesians						
Cluster	Quartz	Kaol.		Exp. dioct.	Ill.		K-fel.	Plag.		Goet.	Magh.	Ilm.	Hem.	Gibb.	Magn.	Anat.		Amph.	Pyrox.		Cal.	Gyps.	∑Phyllo.	∑Fe/Al/Ti-(hydr)ox.
1	98	2		0	0		<1	0		0	0	0	0	0	0	<1		0	0		0	0	2	0
2	85	7		1	4		1	0		0	1	0	0	0	0	<1		0	0		0	0	12	1
3	66	22		2	0		1	0		7	0	<1	1	1	0	<1		0	0		0	0	24	9
4	64	8		2	3		17	4		1	1	0	0	0	0	<1		0	0		0	0	13	2
5	48	24		9	4		2	0		10	0	0	2	0	0	<1		0	0		0	0	37	12
6	47	9		12	9		7	4		7	1	0	0	0	0	<1		3	0		0	0	30	8
7	33	27		13	3		3	2		15	0	2	2	1	0	<1		0	0		0	0	43	20
8	17	1		45	1		9	14		0	4	1	0	1	0	<1		3	0		5	1	46	6
9	10	34		13	0		3	2		18	4	5	3	4	2	<1		0	4		0	0	47	36

A total of 17 minerals were identified across the nine clusters (Table 2). Quartz, phyllosilicates (divided into kaolin, expandable dioctahedral and illitic/micaceous mineral groups), feldspars (K-feldspar and plagioclase) and Fe/Al/Ti-(hydr)oxides (goethite, maghemite, ilmenite, hematite, gibbsite, magnetite and anatase) dominated the mineral compositions (Fig. 5), whilst amphibole (Clusters 6 and 8), pyroxene (Cluster 9), calcite (Cluster 8) and gypsum (Cluster 8) had more limited occurrence. Several of the nine clusters stand out based on the mineralogy of the mean diffractogram (Fig. 5; Table 2). These include the mineralogy of Cluster 1 being almost entirely quartz; Cluster 4 being by far the richest in K-feldspar minerals; Cluster 6 being particularly enriched in illitic/micaceous minerals; Cluster 8 displaying high concentrations of expandable dioctahedral phyllosilicates along with the presence of calcite and gypsum; and Cluster 9 containing high concentrations of Fe/Al/Ti-(hydr)oxides. Together these contrasting soil mineralogies illustrate that the cluster analysis of soil XRPD data yielded nine groups of mineralogically distinct soils, with each group formed of soils from different agro-ecological environments of sub-Saharan Africa (Fig. 3).

**Fig. 6:**
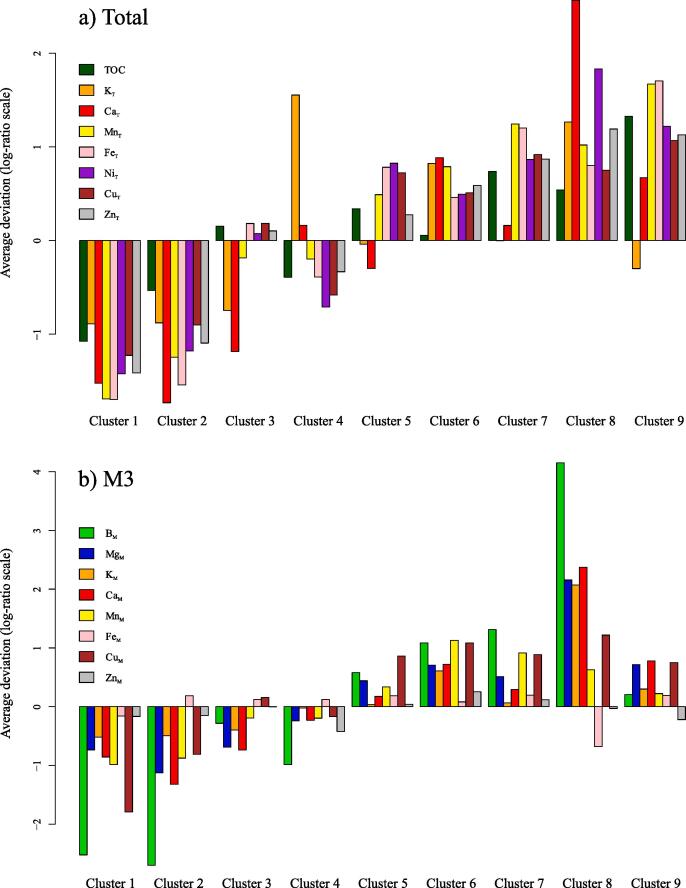
Total (a) and M3 (b) average nutrient concentrations of each cluster expressed as deviation in log-ratio scale from the overall geometric mean of the dataset (i.e. all 935 subsoils; at zero reference line). Values below zero represent average concentrations lower than that for the entire dataset, whilst values above zero represent the opposite.

Nutrient concentrations (total and/or M3 nutrients) also displayed substantial variation between the nine clusters (Table 1). This variation is visualised in Fig. 6 according to the deviation (in log-ratio scale) of each cluster’s geometric mean from that of the entire dataset (Montero-Serrano et al., 2010). Results illustrate general nutrient deficiencies in Clusters 1-4, and enrichments in Clusters 5-9 (Fig. 6). Aside from this broad trend, the mineralogical and geochemical data can be combined to interpret far more specific mineral–nutrient relationships.

#### 4.1.2 Mineral–nutrient relationships

The mean diffractograms of Clusters 1 and 2 represent soils containing 98% and 85% quartz, respectively. The soils within these clusters are consistently deficient in almost all nutrients and micronutrients investigated (Fig. 6; Table 1) with average concentrations consistently near the 10th or 25th percentile of the entire dataset. This deficiency can be explained by the dominance of quartz and kaolin minerals in these soils (Fig. 5; Table 2). Quartz is a notoriously inert mineral that displays negligible contributions to total and extractable soil nutrients (Hardy and Cornu, 2006), whilst kaolinite is not generally considered a significant source of plant nutrients (Dixon and Schulze, 2002). Indeed the consistent nutrient deficiencies of Clusters 1 and 2 suggest that such soils may require special management practices that account for their low nutrient status and potential susceptibility to nutrient leaching in response to fertiliser applications.

Clusters 3 and 4 have almost identical concentrations of quartz (Fig. 5; Table 2), but exhibit distinct K_T_ concentrations (Fig. 6). More specifically, the average K_T_ concentration of Cluster 3 is near the 25th percentile of the dataset, whilst that of Cluster 4 exceeds the 90th percentile (Table 1). These relatively high K_T_ concentrations in Cluster 4 are found to be driven by the enrichment of these soils in K-feldspar minerals (17%; Table 2), which represent a large soil K_T_ reservoir where present (Towett et al., 2015). The contrasting K_T_ concentrations between Clusters 3 and 4 despite their near identical quartz concentrations illustrates how important information can be lost when characterising soil parent material by its silica (i.e. quartz) content alone (Gray et al., 2016).

Clusters 8 and 9 display similar concentrations of both quartz and phyllosilicate mineral components (Fig. 5; Table 2), but differ greatly in M3 nutrient concentrations, with Cluster 8 showing far greater enrichment in B_M_, Mg_M_, K_M_ and Ca_M_ (Fig. 6; Table 1). This difference is driven in part by the contrasting phyllosilicate mineralogies of these clusters, with Cluster 8 dominated by expandable dioctahedral phyllosilicates (45%; Table 2) that can represent substantial sources of adsorbed cations (Velde and Barré, 2009), and Cluster 9 dominated by kaolin minerals (34%; Table 2) that offer limited cation exchange capacities in comparison (Dixon and Schulze, 2002). Further influence of mineralogy on the contrasts in nutrient concentrations between Clusters 8 and 9 relate to the presence of calcite in the soils of Cluster 8 (Table 2), resulting in an alkaline pH (Table 1) that would favour the adsorption of base cations to expandable dioctahedral phyllosilicates (Dixon and Schulze, 2002). Further to the similar concentrations of total phyllosilicate minerals in Clusters 8 and 9, their clay particle size fractions are almost identical (Cluster 8 = 73.4⋇1.18; Cluster 9 = 77.75⋇1.14 Table 1). Together the contrasting nutrient concentrations of Clusters 8 and 9 despite their similar phyllosilicate concentrations (Table 2) and particle size fractions (Table 1) illustrate how important information can be lost when summarising soil clay content as a size fraction (commonplace in soil science; Churchman, 2010) or total phyllosilicate concentration.

A useful attribute of the present dataset is the availability of both total and extractable concentrations of some elements (K, Ca, Mn, Fe, Cu and Zn), which offers a rare insight into how these properties can contrast, and how some of these contrasts are mineralogically driven. In this context, Cluster 4 stands out as having the highest mean K_T_ concentration of all Clusters, but this enrichment is not expressed in the K_M_ concentrations of these soils (Fig. 6; Table 1). This contrast between K_T_ and K_M_ in Cluster 4 illustrates how despite K-feldspar minerals being a major K reserve in these soils, it is not accessible to the M3 extraction, which is in agreement with the notorious resistance of K-feldspar to chemical extractions (Andrist-Rangel et al., 2010, Butler et al., 2018). Further distinctions between total and extractable nutrients are provided in the soils of Cluster 9, which show enrichments in Ca_T_, Mn_T_, Fe_T_, Cu_T_ and Zn_T_ that are not reflected in their M3 equivalents. Thus the total reserves of these nutrients, likely present within the high concentration of Fe/Al/Ti/Mn-(hydr)oxides (Table 2), are not generally accessible to the M3 extraction.

Fe_T_ and Zn_T_ concentrations vary considerably between the nine clusters, but this is not replicated for either Fe_M_ or Zn_M_. The only exception to this lack of variability is the Fe_M_ deficiency of Cluster 8, with these soils having an average Fe_M_ concentration approximately half that of all other clusters (Fig. 6; Table 1). This relative Fe_M_ deficiency of Cluster 8 (with an average near the 10th percentile of the dataset; Table 1) is explained by the alkaline pH of these soils (caused by the presence of calcite; Fig. 5) since the solubility of Fe-(hydr)oxides is particularly dependent upon pH (Lindsay and Schwab, 1982). The consistent lack of variability of Zn_M_ concentrations between the nine mineralogically diverse clusters suggests that neither major soil mineral constituents nor pH are key drivers of Zn_M_ in this case, and further research could seek to identify potentially important mineral sources of phyto-available Zn given recent concerns about Zn deficiencies in crops and humans (Alloway, 2009).

### 4.2 Compositional data analysis

Further mineral–nutrient relationships within the dataset were explored by applying compositional data analysis to the nine clusters (Section 3.2). On a general basis, the PERMANOVA applied to nutrient balances (Sections 3.2.2, 3.2) supports a statistically significant difference in (geometric) mean nutrient compositions between clusters (p<0.0001) for both total and M3 nutrients, reflecting how the contrasting soil mineralogies of the nine clusters yield differences in nutrient compositions.

#### 4.2.1 Balances

**Fig. 7:**
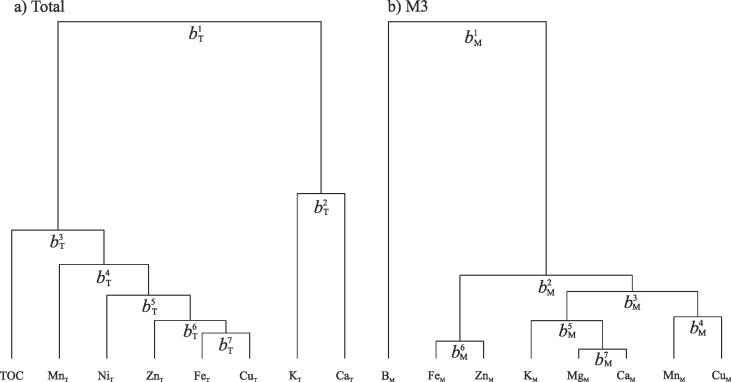
Groupings of the total (a) and M3 (b) nutrient concentration datasets derived from their respective variation matrices (see Tables S1 and S2 and Supplementary Material), used to define meaningful nutrient balances (Table 3).

Dendrograms based on the variation matrices of the total and M3 datasets (Fig. 7; Supplementary Material) show the overall grouping structures of the parts of the respective compositions according to their co-dependence. The dendrogram of the total nutrient dataset presents two clear groupings of co-dependent components (Fig. 7a), the first formed of TOC–Mn_T_–Ni_T_–Zn_T_–Fe_T_–Cu_T_, and the second formed of K_T_–Ca_T_. The groupings were used to define seven balances between the components of the total nutrient dataset (Table 3). The dendrogram of the M3 nutrient dataset presents about four groups of co-dependent components (Fig. 7b). The first of these groups comprises B_M_ alone, which is clearly the most independent element in the M3 dataset. The remaining three groups are comprised of Fe_M_–Zn_M_, Mg_M_–K_M_–Ca_M_, and Mn_M_–Cu_M_. Again these groupings were used to define seven balances between the components of the M3 nutrient dataset (Table 3).

**Table 3:** Nutrient balances for the total and M3 datasets, defined from the overall grouping structure of the variation matrix for each dataset (Fig. 7 and Supplementary Material), along with their contributions to total variance.

Balance	Components	Total variance (%)
*Total*		
$b_{T}^{1}$	TOC–Mn_T_–Fe_T_–Ni_T_–Cu_T_–Zn_T_:K_T_–Ca_T_	40.94
$b_{T}^{2}$	K_T_:Ca_T_	16.64
$b_{T}^{3}$	TOC:Mn_T_–Fe_T_–Ni_T_–Cu_T_–Zn_T_	14.42
$b_{T}^{4}$	Mn_T_:Fe_T_–Ni_T_–Cu_T_–Zn_T_	9.01
$b_{T}^{5}$	Ni_T_:Fe_T_–Cu_T_–Zn_T_	7.54
$b_{T}^{6}$	Zn_T_:Fe_T_–Cu_T_	5.79
$b_{T}^{7}$	Fe_T_:Cu_T_	5.65

*M3*		
$b_{M}^{1}$	B_M_:Mg_M_–K_M_–Ca_M_–Mn_M_–Fe_M_–Cu_M_–Zn_M_	51.75
$b_{M}^{2}$	Fe_M_–Zn_M_:Mg_M_–K_M_–Ca_M_–Mn_M_–Cu_M_	17.47
$b_{M}^{3}$	Mg_M_–K_M_–Ca_M_:Mn_M_–Cu_M_	11.48
$b_{M}^{4}$	Mn_M_:Cu_M_	7.81
$b_{M}^{5}$	K_M_:Mg_M_–Ca_M_	5.61
$b_{M}^{6}$	Fe_M_:Zn_M_	4.21
$b_{M}^{7}$	Mg_M_:Ca_M_	1.68

#### 4.2.2 Principal component biplots

**Fig. 8:**
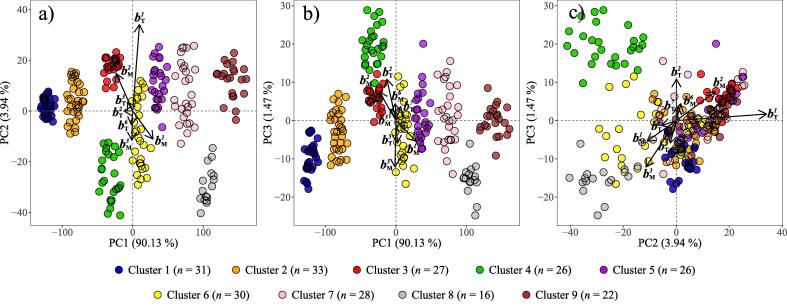
Principal component analysis biplots of the pre-treated and subset (n=239) XRPD data (Section 3.2.3), with the total and M3 nutrient balances (Table 3) added as supplementary variables. Only balances that displayed a discernible vector length are included.

Principal component analysis applied to the subset (n=239; Section 3.2.3) pre-treated XRPD data from the cluster analysis was used to represent the multivariate data in three dimensions (i.e. the first three principal components). These principal components (PCs) together explained 95.54% of total variation of XRPD data (PC1 =90.13%, PC2 =3.94% and PC3 =1.47%), and are plotted in combination with the balances in biplots provided in Fig. 8. More specifically, PC scores in Fig. 8 are represented by point data (coloured by the Cluster factor), whilst the balances are represented as vectors. It is worth noting that since the balances were supplementary variables in the PCA, the PC scores remain solely defined by the XRPD data. In general the biplots illustrate how a range of total and M3 nutrient balances correspond to the principal component scores, and therefore to soil mineralogy. Balances $b_{T}^{1},b_{T}^{2},b_{M}^{1},b_{M}^{2}$ and $b_{M}^{3}$ display the strongest correlations to the PCs, as reflected by their length and proximity to the corresponding PC reference axes presented in Figs. 8a–c.

**Fig. 9:**
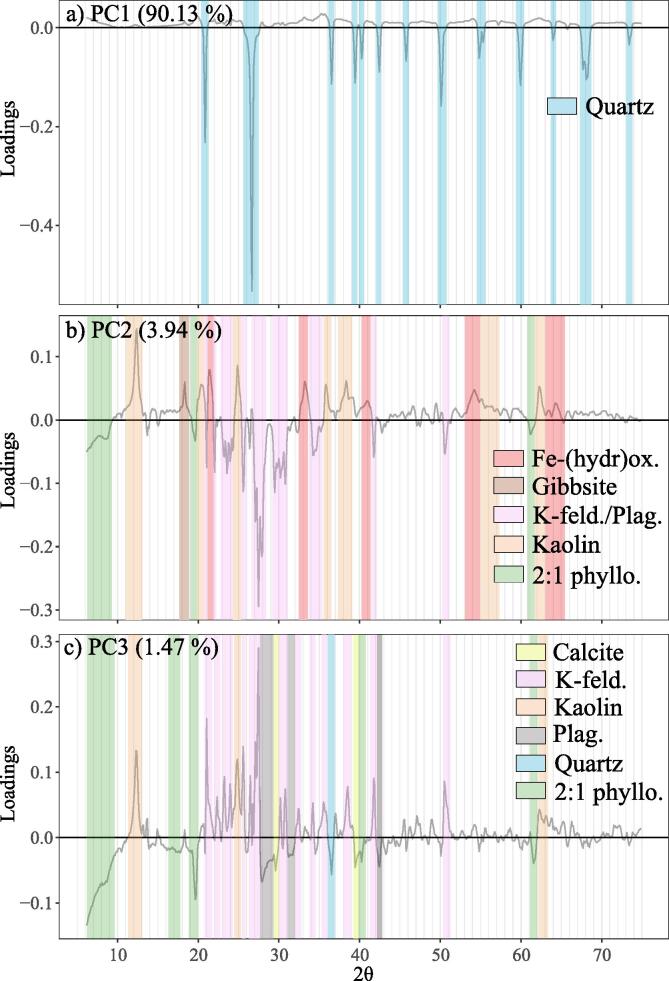
The loadings of PC1 (a), PC2 (b) and PC3 (c) from PCA of the pre-treated and subset (n=239) XRPD data (Section 3.2.3). Highlighted regions represent the soil minerals associated with that specific region of XRPD variables.

The loadings of the three principal component dimensions (Fig. 9) represent the positive or negative contributions of the XRPD variables to the PCs. The Powder Diffraction File database (ICDD, 2019) and the mean diffractograms of the nine clusters were used to assign likely mineral components to regions of notably high or low loading values (Fig. 9) so that mineral–nutrient relationships could be interpreted from Fig. 8.

The loadings of PC1 are dominated by negative values in regions of the XRPD data that are associated with quartz peaks (Fig. 9a). Thus increasing quartz peak intensity would promote decreased PC1 values in Fig. 8. The high proportion of variability accounted for by PC1 (90.13%) is explained by the near ubiquitous presence of quartz in soil (Dixon and Schulze, 2002) along with it being a relatively strong diffractor (Butler et al., 2019). In contrast to PC1, the loadings of PC2 and PC3 represent more subtle features within the diffraction data, accounting for 3.94% and 1.47% of XRPD data variability, respectively. Despite this, the loadings of PC2 and PC3 contain various regions characterised by distinctly positive or negative values that relate to the diffraction features of several common mineral components within the dataset. Namely, positive PC2 loadings are associated with kaolin minerals and Fe/Al-(hydr)oxides, whilst negative PC2 loadings are associated with 2:1 phyllosilicates (i.e. both expandable 2:1 and micaceous 2:1 phyllosilicates), K-feldspar and plagioclase (Fig. 9b). Positive PC3 loadings are associated with K-feldspar and kaolin minerals, whilst negative PC3 loadings are associated with 2:1 phyllosilicates, plagioclase and calcite (Fig. 9c). Together these interpretations of the PC loadings provide a mineralogical meaning for the PCs presented in Fig. 8, allowing further mineral–nutrient relationships to be explored.

#### 4.2.3 Total nutrient balances

Of the seven log-ratio balances defined from the total nutrient dataset (Table 3), $b_{T}^{1}$ and $b_{T}^{2}$ stand out as being related to soil mineralogy based on PCA biplot analysis (Fig. 8).

The balance $b_{T}^{1}$ accounts for 40.94% of variability in the total nutrient dataset (Table 3). This balance partitions the two most distinct groups of co-dependent variables in the total nutrient dataset (Fig. 7), representing TOC–Mn_T_–Fe_T_–Ni_T_–Cu_T_–Zn_T_:K_T_–Ca_T_, and correlates positively with PC2 scores of the XRPD data (Fig. 8). Positive PC2 scores (and increased $b_{T}^{1}$ values) are driven by enrichment of the soils in Fe/Al-(hydr)oxides and kaolin minerals relative to feldspars and 2:1 phyllosilicates. Based on this interpretation of PC2 and estimated chemical compositions of these minerals, it is Fe/Al/Ti-(hydr)oxides that are likely to be the main driver of increased $b_{T}^{1}$ values given their association with Mn, Fe, Ni, Cu and Zn (Bruemmer et al., 1988, Neaman et al., 2008), whilst both 2:1 phyllosilicates and feldspars represent known sources of K_T_ and Ca_T_ (Towett et al., 2015) that are likely to promote decreased $b_{T}^{1}$ values. Soils rich in Fe/Al-(hydr)oxides are often also enriched in Mn-oxides (Neaman et al., 2008), which are difficult to characterise or even identify from complex XRPD data unless especially abundant. Together, Fe/Al/Ti/Mn-(hydr)oxides in soil may also act as strong adsorbents of Ni (Bruemmer et al., 1988), Cu and Zn (Violante et al., 2003), which may partly explain the co-dependence of these elements in $b_{T}^{1}$. Further, the inclusion of TOC in the TOC–Mn_T_–Fe_T_–Ni_T_–Cu_T_–Zn_T_ sub-composition also aligns with the consensus that Fe/Al/Ti/Mn-oxides play an important role in the stabilisation of organic matter in soils and sediments (Lützow et al., 2006, Wagai and Mayer, 2007, Lalonde et al., 2012). In contrast to the geochemistry of Fe/Al-(hydr)oxides, K-feldspar and plagioclase minerals do not usually represent a source of structural or adsorbed transition metal micro-nutrients, and the dominance of feldspars in defining K_T_ and Ca_T_ concentrations likely contributes to the two independent sub-components of $b_{T}^{1}$.

The balance $b_{T}^{2}$ accounts for 17% of variability in the total nutrient dataset, and contrasts to $b_{T}^{1}$ because it partitions the two components of the K_T_–Ca_T_ sub-composition, yielding K_T_:Ca_T_ (Fig. 7; Table 3). Biplots illustrate how $b_{T}^{2}$ positively correlates with PC3 of the XRPD data (Fig. 8). Positive PC3 scores (and increased $b_{T}^{2}$ values) are largely associated with soils rich in K-feldspar and kaolin minerals relative to plagioclase, 2:1 phyllosilicates and calcite, whilst the reverse applies to negative PC3 scores. Pure K-feldspar minerals contain approximately 14% K by weight (Andrist-Rangel et al., 2006) and represent the main K_T_ reservoir in most soils (Towett et al., 2015). Ca-enriched 2:1 phyllosilicates such as Ca-montmorillonite contain up to 2% Ca, plagioclase up to 14% Ca (anorthite), and calcite up to 40% Ca. Based on this information, 2:1 phyllosilicates, plagioclase and calcite could all act as a considerable source of Ca_T_, which is reflected in the Ca_T_ concentration of Cluster 8 being 5 times higher than that of any other cluster (Table 1). As found for $b_{T}^{1}$, this analysis illustrates mineralogical controls on total nutrient concentrations, and highlights how K-feldspar, plagioclase, 2:1 phyllosilicates and calcite control $b_{T}^{2}$.

In summary, total nutrient compositions in the present dataset primarily relate to the concentrations of Fe/Al/Ti/Mn-(hydr)oxides, feldspars, 2:1 phyllosilicates, and calcite. TOC, Mn_T_, Fe_T_, Ni_T_, Cu_T_ and Zn_T_ form a group of co-dependent variables that reflect how Fe/Al/Ti/Mn-(hydr)oxides are the main host of these transition metal micro-nutrients in soil, and that these minerals have a tendency to promote the stabilisation of soil organic matter (Wagai and Mayer, 2007, Zhao et al., 2016). K_T_ and Ca_T_ concentrations are largely independent of the TOC–Mn_T_–Fe_T_–Ni_T_–Cu_T_–Zn_T_ sub-composition and primarily relate to K-feldspar and plagioclase minerals along with contributions from 2:1 phyllosilicates and calcite.

#### 4.2.4 M3 nutrient balances

Of the seven log-ratio balances defined from the M3 nutrient dataset (Table 3), $b_{M}^{1},b_{M}^{2}$ and $b_{M}^{3}$ are found to have specific relationships to soil mineralogy based on PCA biplot analysis (Fig. 8).

B_M_ is the most independent nutrient of the M3 dataset (Fig. 7) and is partitioned from all other M3 nutrients in $b_{M}^{1}$ (B_M_:Mg_M_–K_M_–Ca_M_–Mn_M_–Fe_M_–Cu_M_–Zn_M_), which accounts for 51.75% of variability in the M3 dataset (Table 3). This independence is primarily controlled by the enrichment of B_M_ in the soils of Cluster 8, which have a mean B_M_ concentration 17 times higher than that of any other cluster (Table 1). Thus $b_{M}^{1}$ is consistently associated with Cluster 8 in the PCA biplots (Fig. 8), driven by low quartz concentrations (positive PC1 scores), enrichment in 2:1 phyllosilicates (negative PC2 and PC3 scores) and the presence of calcite (negative PC3 scores). The notable enrichment of Cluster 8 in 2:1 phyllosilicates, especially expandable dioctahedral minerals, combined with the presence of calcite (Table 2), therefore act to create soils with high B_M_ concentrations. In this context the presence of calcite explains the alkaline pH of the soils of Cluster 8 (pH = 8.02±0.90; Table 1), which would affect B adsorption to 2:1 phyllosilicates (Karahan et al., 2006). More specifically, aqueous B_M_ concentrations are driven by the equilibrium reaction of boric acid (H_3_BO_3_):

$$H_{3}{\mathrm{BO}}_{3}+H_{2}O⇌B{\left(\mathrm{OH}\right)}_{4}^{-}+H^{+}\text{.}$$

Below pH 7, H_3_BO_3_ predominates, whilst alkaline solutions favour $B{\left(\mathrm{OH}\right)}_{4}^{-}$ (Russell and Wild, 1988). In alkaline conditions (pH >8; Karahan et al., 2006) 2:1 phyllosilicates have a high capacity for $B{\left(\mathrm{OH}\right)}_{4}^{-}$ adsorption, whilst at pH<7 B is readily leached from the soil which can result in B deficiencies (Gupta et al., 1985). The alkaline pH of the soils of Cluster 8 would promote adsorption of B by the expandable dioctahedral minerals in the form of $B{\left(\mathrm{OH}\right)}_{4}^{-}$ (Karahan et al., 2006). All other clusters had similarly acidic pH’s on average (Table 1), limiting the potential for $B{\left(\mathrm{OH}\right)}_{4}^{-}$ adsorption by phyllosilicates. Together this not only highlights the combined importance of calcite, phyllosilicate minerals and pH on soil B availability, but also suggests that the M3 extraction results in the desorption of $B{\left(\mathrm{OH}\right)}_{4}^{-}$ from expandable dioctahedral phyllosilicates.

The balance $b_{M}^{2}$ represents the contrast of Fe_M_–Zn_M_:Mg_M_–K_M_–Ca_M_–Mn_M_–Cu_M_ that accounts for 17.47% of variability in the M3 nutrient dataset. It is worth noting that of all M3 nutrients, Fe_M_ and Zn_M_ display the least variation between the nine clusters (Fig. 6), therefore drivers of variation in $b_{M}^{2}$ will primarily relate to changes within the Mg_M_–K_M_–Ca_M_–Mn_M_–Cu_M_ sub-composition. $b_{M}^{2}$ displays positive correlations with PCs 2 and 3, and negative correlations with PC1. Increased $b_{M}^{2}$ values are therefore promoted by soils enriched in quartz, Fe/Al-(hydr)oxides, K-feldspar, and kaolin relative to 2:1 phyllosilicates, plagioclase and calcite. Of the minerals within this list, quartz would not represent a source of any M3 nutrients (Hardy and Cornu, 2006), whilst structural Ca and K in plagioclase and K-feldspars, respectively, would not contribute considerably to the M3 nutrient concentrations since these framework silicates are particularly resistant even to quite aggressive chemical extractants (Andrist-Rangel et al., 2010, Reimann et al., 2014, Butler et al., 2018). Of the remaining minerals driving changes in $b_{M}^{2}$, 2:1 phyllosilicates represent the most likely source of elements within the Mg_M_–K_M_–Ca_M_–Mn_M_–Cu_M_ sub-composition. It is therefore considered that $b_{M}^{2}$ represents how 2:1 phyllosilicates are less likely to represent a source of Fe_M_ or Zn_M_ than they are of Mg_M_, K_M_, Ca_M_, Mn_M_ or Cu_M_.

The balance $b_{M}^{3}$ partitions the Mg_M_–K_M_–Ca_M_–Mn_M_–Cu_M_ sub-component of $b_{M}^{2}$, representing Mg_M_–K_M_–Ca_M_:Mn_M_–Cu_M_ and accounting for 11.48% of variability in the M3 nutrient dataset. It is found that $b_{M}^{3}$ correlates negatively with PCs 2 and 3 (Fig. 8). Negative correlation of $b_{M}^{3}$ with PC2 suggests soils enriched in 2:1 phyllosilicates and feldspar minerals relative to kaolin and Fe/Al-(hydr)oxides favour increased $b_{M}^{3}$ values. Similarly the negative correlation of $b_{M}^{3}$ with PC3 is also driven by enrichment of the soil in 2:1 phyllosilicates along with the presence of calcite. Given these mineral–nutrient relationships, soils in Clusters 6 (enriched in 2:1 phyllosilicates) and 8 (enriched in 2:1 phyllosilicates and calcite) display the highest $b_{M}^{3}$ values (Fig. 8). Since PC2 scores are not affected by calcite and yet still relate to $b_{M}^{3}$, the data suggest that 2:1 phyllosilicates are more likely to represent sources of nutrients within the Mg_M_–K_M_–Ca_M_ sub-composition than the Mn_M_–Cu_M_ sub-composition - in agreement with most literature highlighting 2:1 phyllosilicates as sources of base cations (Velde and and Barré, 2009, Churchman, 2010, Singh and Schulze, 2015). Therefore, assuming that 2:1 phyllosilicates represent the main source of M3 nutrient concentrations, $b_{M}^{2}$ and $b_{M}^{3}$ together suggest an approximate sequence of co-dependent soil nutrients that are associated with these minerals of Mg_M_–K_M_–Ca_M_ > Mn_M_–Cu_M_ > Fe_M_–Zn_M_.

In summary, whilst Fe/Al/Ti/Mn-(hydr)oxides and feldspar minerals are key drivers of total soil nutrient concentrations in the present dataset (Section 4.2.3), it is 2:1 phyllosilicates that represent the main source of all M3 extractable nutrients except for Fe_M_ and Zn_M_. Since 2:1 phyllosilicates are therefore not likely to represent a source of Fe_M_ or Zn_M_ in soils investigated here, potential deficiencies in these elements must be accounted for by other organic or mineral soil components.

### 4.3 Future prospects

The mineral–nutrient relationships defined here from the cluster analysis and compositional methods illustrate how mineralogy is the key driver of soil nutrient concentrations in African soils. With increasing availability of geo-referenced soil XRPD datasets (Hillier and Butler, 2018) and associated geochemical data, further data-driven research could progress towards classifying soil nutrient statuses from XRPD measurements via the use of cluster analysis or other data-driven methods. These ‘Digital Mineralogy’ approaches could aid in defining mineralogically tailored nutrient management schemes that can account for the full suite of essential plant nutrients. Doing so would help land users avoid the risks of excessive fertiliser use (Sebilo et al., 2013) and nutrient mining (Jones et al., 2013), and aid in tackling human micro-nutrient deficiencies (Alloway, 2009).

## 5 Conclusions

Cluster analysis of soil XRPD data was used to define nine mineralogically distinct clusters from the soil mineralogy continuum. Each of the nine clusters derived from XRPD data was comprised of mineralogically similar soils from different agro-ecological environments of sub-Saharan Africa. Despite this spatial variation, meaningful differences between clusters were found for both the total and M3 nutrient compositions. Quartz and kaolin minerals did not show a notable contribution to any of the total or M3 nutrient concentrations analysed. Feldspar minerals were found to be major sources of K_T_ (K-feldspar) and Ca_T_ (plagioclase), but did not directly contribute to K_M_ or Ca_M_. Fe/Al/Ti/Mn-(hydr)oxides were found to be associated with TOC concentrations as reflected in the co-dependence of TOC, Mn_T_, Fe_T_, Ni_T_, Cu_T_ and Zn_T_ sub-composition. Compared to the contribution of Fe/Al/Ti/Mn-(hydr)oxides to total nutrient concentrations, their importance as a source of nutrients accessible to the M3 extraction was found to be limited. All M3 nutrient concentrations except for Fe_M_ and Zn_M_ were primarily driven by 2:1 phyllosilicates, namely expandable dioctahedral and illitic/micaceous minerals. The importance of 2:1 phyllosilicates as a source of phyto-available nutrients suggests that the relative abundance of these minerals could provide a powerful indicator of the inherent fertility in African soils.

The interpreted soil mineralogy–nutrient relationships highlight how the soil mineral composition drives soil nutrient concentrations and their phyto-availability. As such, characterising soils based on their silica (e.g. basic vs acidic) and/or clay (e.g. soil texture) components alone acts to over-simplify the intricacies of mineral contributions to soil nutrients.

This is the first application of cluster analysis to soil XRPD data, from which it is evident that its use in combination with compositional data analysis methods can allow for detailed interpretation of soil mineral–nutrient relationships. Information encoded within soil XRPD data is thus inherently related to the total and/or extractable concentrations of B, Mg, K, Ca, Mn, Fe, Ni, Cu and Zn. Data-driven analysis of soil XRPD data can therefore be used to extract this information in new detail.

## Declaration of Competing Interest

The authors declare that they have no known competing financial interests or personal relationships that could have appeared to influence the work reported in this paper.
