# Supplementary material for: Mineral–nutrient relationships in African soils assessed using cluster analysis of X-ray powder diffraction patterns and compositional methods
Source: Geoderma. 2020 Oct 1;375:114474. doi: 10.1016/j.geoderma.2020.114474 (PMC7386901; doi:10.1016/j.geoderma.2020.114474)
Supplement: Supplementary data [file mmc1.pdf]

Supplementary Material: Compositional analysis of nutrient concentration compositions using log-ratio balances based on co-dependence

Most ordinary statistical methods are designed for real-valued variables and focus on the absolute magnitude of the measurements as basic input for comparison between samples. However, compositions consist of inter-related collections of parts of a whole within which values are relative to each other; commonly expressed in units such as percentages, parts per million, weight percent or similar. Without loss of generality, when a practitioner closes the data to add up to a constant (e.g. 1 when expressing the data in proportions) the composition is equivalently represented on a so-called unit simplex. The construction and analysis of log-ratio transformations involving a one-to-one mapping from the simplex to the ordinary real space have become the mainstream approach to deal with data carrying relative information (Pawlowsky-Glahn et al., 2015). Log-ratios are real-valued and this generally enables the use of ordinary data analysis techniques on them, with the possibility of transferring results and conclusions back to be expressed in terms the original compositions. This type of data transformation additionally guarantees that results do not change with changes in the units of measurement used (e.g. if data were rescaled from mg kg^{-1} to proportions) or depending on whether the full composition or only a subset of its parts (a sub-composition) is of interest, avoiding then conflicting interpretations when using different scaling procedures. The issues with data representing parts of a whole and compositional methods have been known for long in the geological sciences (see e.g. Buccianti et al. (2006) and Grunsky and de Caritat (2019)), however in recent decades they have found applications in a wide range of areas, from modern molecular biology and epidemiology to economics and social sciences. Recent applications in soil science can be found in, for example, Reimann et al. (2012), Abdi et al. (2015) and Neiva et al. (2019).

We use in this work so-called isometric log-ratio (ilr) coordinates. In particular, a family of ilr-coordinates known as *balances*, which represent the relative importance or weight of one part or group of parts of the composition with respect to another part or group of parts as summarised by their corresponding geometric means (Pawlowsky-Glahn et al., 2015). A collection of $D-1$ balances is obtained from a D-part composition. Formally, given a composition $\mathbf{x} = (x_i, \ldots, x_D)$, a procedure to construct balances b_i is based on a sequential binary partition which produces contrasts between two subsets of parts as

$$b_i = \sqrt{\frac{r_i s_i}{r_i + s_i}} \ln \frac{(\prod_{k=1}^{r_i} x_{ik}^{+})^{1/r_i}}{(\prod_{k=1}^{s_i} x_{ik}^{-})^{1/s_i}}, \quad i = 1, \ldots, D-1,$$

where x_{ik}^{+} and x_{ik}^{-} refer to the subsets of r_i and s_i parts of $\mathbf{x}$ going, respectively, into the $+$ (numerator) and $-$ (denominator) groups. The multiplicative factor preceding the log-ratio term is a normalisation factor required to guarantee orthogonality of the balances.

In accordance with the relative scale of the data, the co-dependence or association between parts is determined in terms of proportionality between pairs of parts, instead of using the ordinary Pearson correlation measure. Following (Aitchison, 1986), proportionality was measured by the matrix of log-ratio variances $\mathbf{T} = [t_{ij}]_{D\times D}$, the so-called variation matrix, where $t_{ij} = \mathrm{var}(\log(x_i/x_j))$, with $i, j = 1, \ldots, D$. A log-ratio variance t_{ij} which is close to 0 indicates that the two components x_i and x_j are nearly proportional (highly co-dependent); that is, their log-ratio is nearly constant. Tables S1 and S2 show the variation matrices obtained for the total and M3 datasets respectively. Note that the variance (var) was computed here using the mean absolute deviation (MAD) as a

Table S1: Variation matrix for the total nutrient concentration dataset.

	TOC	K_T	Ca_T	Mn_T	Fe_T	Ni_T	Cu_T	Zn_T
TOC	0.0000	2.1385	2.2395	1.0818	0.6213	1.1334	0.7551	0.7700
K_T	2.1385	0.0000	1.3831	2.0165	2.2205	2.0169	1.7275	1.4977
Ca_T	2.2395	1.3831	0.0000	1.4229	1.8569	1.4115	1.6666	1.3284
Mn_T	1.0818	2.0165	1.4229	0.0000	0.4946	0.9090	0.6420	0.5302
Fe_T	0.6213	2.2205	1.8569	0.4946	0.0000	0.5053	0.2127	0.2952
Ni_T	1.1334	2.0169	1.4115	0.9090	0.5053	0.0000	0.3119	0.5298
Cu_T	0.7551	1.7275	1.6666	0.6420	0.2127	0.3119	0.0000	0.2950
Zn_T	0.7700	1.4977	1.3284	0.5302	0.2952	0.5298	0.2950	0.0000

Table S2: Variation matrix for the M3 nutrient concentration dataset.

	B_M	Mg_M	K_M	Ca_M	Mn_M	Fe_M	Cu_M	Zn_M
B_M	0.0000	4.1007	3.5405	4.2299	6.8071	13.2442	5.9461	10.4205
Mg_M	4.1007	0.0000	0.8315	0.2772	1.4220	1.6855	1.0282	1.6573
K_M	3.5405	0.8315	0.0000	1.1261	2.0470	1.2512	1.6436	1.3436
Ca_M	4.2299	0.2772	1.1261	0.0000	1.4994	1.6542	1.2192	1.8680
Mn_M	6.8071	1.4220	2.0470	1.4994	0.0000	2.4062	1.2453	1.9956
Fe_M	13.2442	1.6855	1.2512	1.6542	2.4062	0.0000	1.5801	0.5122
Cu_M	5.9461	1.0282	1.6436	1.2192	1.2453	1.5801	0.0000	1.3298
Zn_M	10.4205	1.6573	1.3436	1.8680	1.9956	0.5122	1.3298	0.0000

robust counterpart to the ordinary variance to downplay the influence of some outlying samples.

For example, the variation matrix for the M3 dataset (Table S2) shows that Ca_M and Mg_M on the one hand and Fe_M and Zn_M on the other hand have the strongest proportionality relationships (lowest log-ratio variance value equal to 0.2772 and 0.5122 respectively); whereas B_M clearly exhibits the weakest relationships with all the other components (high values in all log-ratio variances involving it). This structure of relationships can be visually represented in two dimensions using a compositional biplot (Figure S1) obtained by conducting principal component analysis (PCA) after applying a centred log-ratio (clr) transformation of the data (Aitchison and Greenacre, 2002). The clr-variables $\mathbf{y} = \text{clr}(\mathbf{x})$ are computed as

$$y_i = \log\left(\frac{x_i}{(\prod_{k=1}^{D} x_k)^{1/D}}\right), \quad i = 1, \ldots, D.$$

The relationships between components in Figure S1 are reflected in the length of the links between rays for the different parts. The shorter the link between two arrowheads the higher the co-dependence (proportionality) between the corresponding parts. This is in agreement with the variation matrices (although note that the biplot is a 2-dimensional representation and some information is lost; in this case the biplot based on the first two principal components accounts for 71% and 67% of the total variability in the total and M3 datasets respectively). For the M3 dataset, the co-dependences Ca_M–Mg_M and Fe_M-Zn_M are clearly illustrated. Note that the rays for these two pairs lay nearly perfectly on a line, so they form a one-dimensional pattern of variation. Moreover, Mn_M, Fe_M and B_M are the longest rays and point towards different directions, with their links approximately forming 90 degrees angles between them, hence indicating that a high portion of the total variability between samples is related to their abundances. For the total nutrient dataset, the samples appear fairly homogeneously spread, with variability mostly driven by Ca_T, K_T, TOC and Mn_T.

The information in the variation matrix $\mathbf{T}$ was used in this work as input to perform hierarchical clustering of variables (R-mode Ward method), and the derived overall grouping structure of the parts (according to their proportionality) was displayed in the associated dendrograms. They are

shown for the total and M3 datasets in Figure 7 of the manuscript. These groupings were used here to define data-driven balances between nutrient concentrations, with the split at each node of the dendrogram determining the parts going into the numerator (left-hand branch) and denominator (right-hand branch) of the log-ratio term of each balance (b^i_{T} or b^i_{M}, $i = 1, \ldots, 7$). Thus, for the M3 dataset for instance, b^1_{M} is given by

$$b^1_{\mathrm{M}} = \sqrt{\frac{7}{8}} \ln \frac{\mathrm{B_M}}{(\mathrm{Fe_M} \cdot \mathrm{Zn_M} \cdot \mathrm{K_M} \cdot \mathrm{Mg_M} \cdot \mathrm{Ca_M} \cdot \mathrm{Mn_M} \cdot \mathrm{Cu_M})^{1/7}}$$

which represents a contrast between $\mathrm{B_M}$ and the other nutrients (summarised by their geometric mean), b^3_{M} represents $\mathrm{K_M}$–$\mathrm{Mg_M}$–$\mathrm{Ca_M}$ against $\mathrm{Mn_M}$–$\mathrm{Cu_M}$,

$$b^3_{\mathrm{M}} = \sqrt{\frac{6}{5}} \ln \frac{(\mathrm{K_M} \cdot \mathrm{Mg_M} \cdot \mathrm{Ca_M})^{1/3}}{(\mathrm{Mn_M} \cdot \mathrm{Cu_M})^{1/2}},$$

the balance b^5_{M} is $\mathrm{K_M}$ against $\mathrm{Mg_M}$–$\mathrm{Ca_M}$,

$$b^5_{\mathrm{M}} = \sqrt{\frac{2}{3}} \ln \frac{\mathrm{K_M}}{(\mathrm{Mg_M} \cdot \mathrm{Ca_M})^{1/2}},$$

and so on. Note that a balance is equal to zero in equilibrium, so the sign of the balance indicates the predominance of one subset of components versus the other. Moreover, these balances account for decreasing amounts of total variability in the dataset. Thus, for the M3 dataset, they account for 51.75%, 17.47%, 11.48%, 7.81%, 5.61%, 4.21% and 1.68% from b^1_{M} to b^7_{M} respectively. In the total dataset, these contributions are 40.94%, 16.64%, 14.42%, 9.01%, 7.54%, 5.79% and 5.65% from b^1_{T} to b^7_{T} respectively. Hence, most variability is explained by the first balance and then the explained variability decreases gradually.

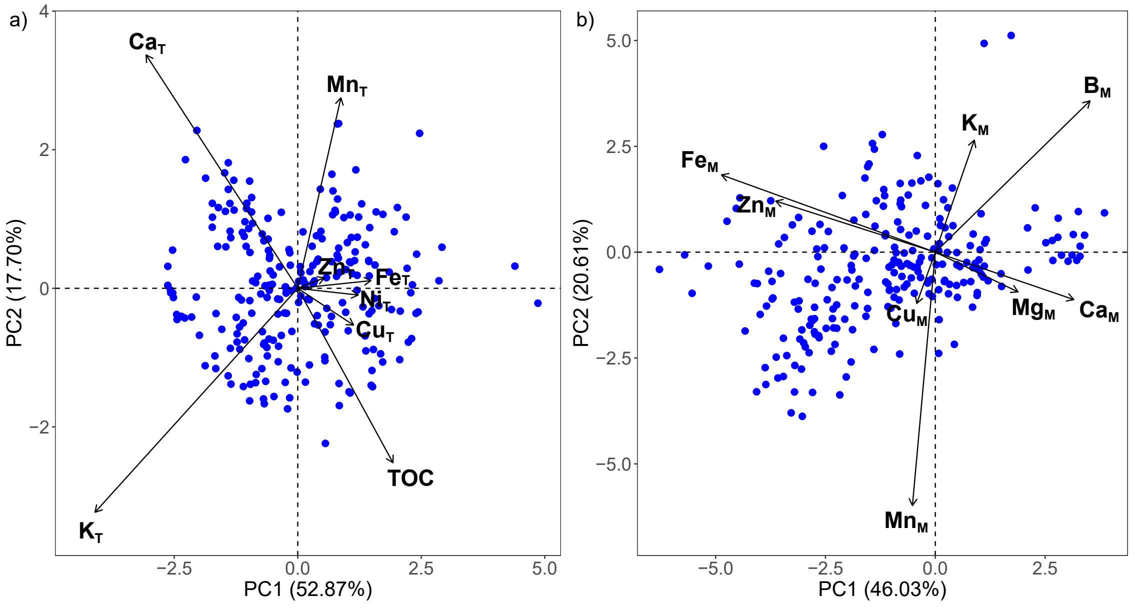

Figure S1: Compositional biplots of a) total and b) M3 nutrient concentration datasets.

The collections of log-ratio balances for the total and M3 nutrient concentration compositions are defined on the ordinary real space and ordinary statistical techniques can be applied on them. Thus, the multivariate analysis of variance described in the manuscript was conducted on balances. Finally, note that balances, and ilr-coordinate systems in general, can be defined in infinitely many ways, with them being just orthogonal rotations from each other from a geometrical point of view.

Hence, it is important to verify that a particular data analysis method or model is invariant to those rotations, which enables to use an arbitrary choice of ilr-coordinates to obtain the required output. Even so, in many practical settings it is preferable to tailor the balance representation according to a meaningful criterion for the benefit of interpretability. For example, balances driven by information contained in the data (as we did above based on the co-dependency structure between elements) or by the scientific questions to represent particular relationships of interest. It can be checked that the statistical methods used in this study are invariant to orthogonal rotations of ilr-coordinates.

References

Abdi, D., Cade-Menun, B. J., Ziadi, N., and Parent, L.-É. (2015). Compositional statistical analysis of soil 31p-nmr forms. *Geoderma*, 257:40–47.

Aitchison, J. (1986). *The statistical analysis of compositional data.* Monographs on statistics and applied probability. Chapman and Hall.

Aitchison, J. and Greenacre, M. (2002). Biplots of compositional data. *Journal of the Royal Statistical Society: Series C (Applied Statistics)*, 51(4):375–392.

Buccianti, A., Mateu-Figueras, G., and Pawlowsky-Glahn, V. (2006). *Compositional data analysis in the geosciences: From theory to practice.* Geological Society of London.

Grunsky, E. and de Caritat, P. (2019). State-of-the-art analysis of geochemical data for mineral exploration. *Geochemistry: Exploration, Environment, Analysis*, 20(2):217–232.

Neiva, A., Albuquerque, M., Antunes, I. M. H. R., Carvalho, P., Santos, A., Boente, C., Cunha, P., Henriques, S., and Pato, R. (2019). Assessment of metal and metalloid contamination in soils trough compositional data: the old Mortórios uranium mine area, central Portugal. *Environmental geochemistry and health*, 41:2875–2892.

Pawlowsky-Glahn, V., Egozcue, J., and Tolosana-Delgado, R. (2015). *Modeling and Analysis of Compositional Data.* Statistics in Practice. Wiley.

Reimann, C., Filzmoser, P., Fabian, K., Hron, K., Birke, M., Demetriades, A., Dinelli, E., Ladenberger, A., and Team, T. G. P. (2012). The concept of compositional data analysis in practice – total major element concentrations in agricultural and grazing land soils of europe. *Science of the total environment*, 426:196–210.
